# Sustained effect of leukocytapheresis/granulocytapheresis versus anti-human TNF-α monoclonal antibody on ulcerative colitis: A 2-year retrospective study

**DOI:** 10.1097/MD.0000000000033368

**Published:** 2023-04-21

**Authors:** Masahiro Sakai, Koichi Hayashi, Tomoyuki Ito, Haruka Otani, Yuya Mori, Shinsuke Ito, Keita Endo, Hiroto Matsuda, Kaede Yoshino, Koichi Kitamura, Eiji Kubota, Yasuaki Motomura, Yasuhiro Suzuki, Shigeki Fujitani, Toshihiko Suzuki

**Affiliations:** a Department of Nephrology, Endocrinology and Diabetes, Tokyo Bay Urayasu Ichikawa Medical Center, Urayasu, Chiba, Japan; b Department of Emergency and Critical Care Medicine, St. Marianna University Yokohama Seibu Hospital, Yokohama, Kanagawa, Japan; c Department of Pharmacy, Tokyo Bay Urayasu Ichikawa Medical Center, Urayasu, Chiba, Japan; d Department of Nephrology and Hypertension, Keiyu Hospital, Yokohama, Kanagawa, Japan; e Department of Nephrology and General Medicine, Shizuoka Red Cross Hospital, Shizuoka, Shizuoka, Japan; f Department of Gastroenterology, Tokyo Bay Urayasu Ichikawa Medical Center, Urayasu, Chiba, Japan; g Department of Medical Engineering, Tokyo Bay Urayasu Ichikawa Medical Center, Urayasu, Chiba, Japan; h Department of Emergency and Critical Care Medicine, St. Marianna University School of Medicine, Kawasaki, Kanagawa, Japan.

**Keywords:** anemia, biological preparation, clinical activity index, cost-effectiveness, cytapheresis, platelet, ulcerative colitis

## Abstract

Although anti-tumor necrosis factor-α monoclonal antibody biological preparations (BP) agents are widely used as an established treatment tool for refractory ulcerative colitis (UC), whether leukocytapheresis/granulocytapheresis (L/G-CAP) has similar beneficial impact on the disease activity remains undetermined. Furthermore, the costs defrayed for the treatment with these 2 modalities have not been compared. We retrospectively evaluated whether L/G-CAP offered sustained beneficial effects over 2-year period. The patients who had moderately to severely active UC (Rachmilewitz clinical activity index (CAI) ≧ 5) and were treated with a series (10 sessions) of L/G-CAP (n = 19) or BP (n = 7) as an add-on therapy to conventional medications were followed. Furthermore, the cost-effectiveness pertaining to the treatment with L/G-CAP and BP was assessed over 12 months. At baseline, L/G-CAP and BP groups manifested similar disease activity (CAI, L/G-CAP; 7.0 [6.0–10.0], BP; 10.0 [6.0–10.0], *P* = .207). The L/G-CAP and BP treatment suppressed the activity, with CAI 1 or less attained on day 180. When the L/G-CAP group was dichotomized into L/G-CAP-high and L/G-CAP-low group based on CAI values (≥3 or < 3) on day 365, CAI was gradually elevated in L/G-CAP-high group but remained suppressed in L/G-CAP-low group without additional apheresis for 2 years. Anemia was corrected more rapidly and hemoglobin levels were higher in BP group. The cost of the treatment with L/G-CAP over 12 months was curtailed to 76% of that with BP (1.79 [1.73–1.92] vs 2.35 [2.29–3.19] million yen, *P* = .028). L/G-CAP is as effective as BP in a substantial number of patients over 2 years. The cost for the treatment of UC favors L/G-CAP although the correction of anemia may prefer BP. Thus, L/G-CAP can effectively manage the disease activity with no additional implementation for 2 years although further therapeutic modalities might be required in a certain population with high CAI observed on day 365.

## 1. Introduction

Ulcerative colitis (UC) is a chronic inflammatory bowel disease with repeated relapses and remissions and mainly affects the mucosa of the colon. Although the etiology of UC remains undetermined fully and is thought to be multifactorial,^[1]^ recruitment of circulating leukocytes to the inflamed mucosa through the release of chemoattractant presumably constitutes a critical process for amplification of the inflammatory response in UC.^[1,2]^ Conventionally, mesalazine, corticosteroids, and other immunomodulators have been applied solely or in combination to the treatment of UC, depending on the severity of the disease.^[1,3]^ In highly active cases, however, the effectiveness of these therapeutic tools in suppressing the disease activity and inducing the remission appears unsatisfactory,^[4]^ which thus indicates the need for additional therapeutic modalities.

Recent therapeutic strategies for UC are expanding since the application of antitumor necrosis factor (TNF)-α monoclonal antibody agents to moderately to severely active UC,^[5–7]^ and this tool, that is, a biological preparation (BP), has now been established as a standard therapy for moderately to severely active UC. Moreover, leukocytapheresis/granulocytapheresis (L/G-CAP), which removes activated leukocytes from the peripheral blood through an extracorporeal circulation, has been introduced into the treatment of active UC^[8–10]^ and the Japanese diagnostic criteria and treatment guidelines for ulcerative colitis and Crohn disease (http://www.ibdjapan.org/pdf/doc15.pdf) also address L/G-CAP as a treatment option. Although these 2 therapeutic strategies confer greater benefit, it has not been determined thus far whether L/G-CAP is as effective as BP in suppressing the disease activity and inducing the remission of UC. Of note, both L/G-CAP and BP therapies entail more enormous cost than conventional therapies,^[11–13]^ which hence arouses financial concern, for example, economic burden on individuals and health care organizations. Recently, Kobayashi et al^[14]^ demonstrated sustained posttreatment effects of L/G-CAP that had been conducted 1 year before evaluation. Furthermore, Takayama et al^[15]^ showed that among L/G-CAP-treated patients, 49% of the cases achieved clinical remission after a single course of the apheresis during more than 3-year follow-up period. Because the treatment with BP requires the regular administration based on the standard protocol, it appears critically important to evaluate the sustained efficacy of L/G-CAP and further to characterize the temporal profiles of inflammatory markers, anemia, and renal function which may be affected by multiple confounding factors, including the disease activity per se and the underlying medications. Nevertheless, there have been no studies reported so far that compare the long-term effects of L/G-CAP with those of BP therapies in maintaining the remission of UC.

We therefore evaluated retrospectively the efficacy of L/G-CAP and BP in suppressing the activity of UC as well as the cost-effectiveness and adverse effects pertaining to the treatment with these therapies. More importantly, whether the L/G-CAP therapy retained long-lasting effects was assessed based on the 2-year observations on the disease activity and inflammatory parameters.

## 2. Materials and methods

This study is a retrospective analysis evaluating the long-term efficacy of L/G-CAP and anti-TNF-α monoclonal antibody agents on the disease activity during a total of 2 years, and also assessing the costs of the treatment for UC over 12-month period (UMIN trial No. 000036954). The study was approved by the Ethics Committee of Tokyo Bay Urayasu Ichikawa Medical Center (Chiba, institutional approval No. 376) and Keiyu Hospital (Yokohama, Kanagawa, institutional approval No. R3-26) and was conducted in accordance with the Declaration of Helsinki. Information from medical records was anonymized and de-identified prior to final analysis. The informed consent was waived because of retrospective nature of this study.

## 3. Study population

We enrolled 26 patients who had been diagnosed as UC in Tokyo Bay Urayasu Ichikawa Medical Center (Urayasu, Chiba) or Keiyu Hospital (Yokohama, Kanagawa) between April 2013 and December 2018. Endoscopic and histological diagnosis of UC was made in all patients, thus excluding indeterminate colitis. The patients had moderate to severe activity of UC, presenting with Rachmilewitz clinical activity index (CAI) 5 or greater.^[16]^ Almost all patients had been treated with conventional therapies, including mesalazine and corticosteroids, but exhibited steroid-resistance or -dependency, which indicated the need for additional therapeutic modalities. Nineteen patients were thus treated with L/G-CAP and the results were compared with those obtained in 7 patients treated with BP as reference data.

## 4. Protocols

### 4.1. One year evaluation of treatment efficacy and cost-effectiveness

L/G-CAP was performed twice a week until the disease activity was stabilized, and a total of 10 sessions were conducted. Cellsorba Ex (Asahi Kasei Medical Co., Ltd, Tokyo) or Adacolumn (JIMRO, Takasaki, Japan) (https://www.adacyte.com/wp-content/uploads/2021/02/adacolumn-instructions-for-use.pdf) was used for apheresis at a blood flow rate of 30 to 50 mL/minutes and at a blood processing volume of 30 mL/kg body weight or more. Then, the conditions of the patients enrolled were followed for 1 year.

Regarding the treatment with BP, either infliximab or adalimumab was administered according to the standard protocol. In brief, infliximab was given intravenously at 5 mg/kg and was repeated at 2 and 6 weeks, and every 8 weeks thereafter. Adalimumab was administered subcutaneously at 160 mg at week 0, 80 mg at week 2, and then 40 mg every 2 weeks. Either drug protocol was iterated during the study period.

The disease activity of UC was determined by evaluating inflammatory markers, including white blood cell/platelet counts, blood hemoglobin levels, C-reactive protein (CRP), erythrocyte sedimentation rate (ESR), and CAI, along with physical manifestations (e.g., bloody stool, abdominal cramps and fever) during the 12 months of the study period.

The expense incurred during the 12-month treatment was estimated based on the costs of conventional drugs (mesalazine, prednisolone, azathiopurine), BP (infliximab, adalimumab) and apheresis devices (Cellsorba Ex/Adacolumn). Total expenses were assessed by adding together the costs of the drugs and the devices used in each patient. The efficacy and the costs of the treatment was evaluated at 1, 3, 6, and 12 months from the initiation of the L/G-CAP or the BP therapy, and the results were compared between these 2 therapies.

### 4.2. Evaluation of 2-year sustained efficacy of L/G-CAP

Because 1 series (i.e., 10 sessions) of L/G-CAP was performed at the initiation of the study and conventional drug administration was continued thereafter, whether the efficacy of a series of the apheresis was sustained for an additional year was evaluated. CAI was serially followed for assessment of the disease activity, and various blood parameters, including blood hemoglobin, platelet counts, serum creatinine, and estimated glomerular filtration rate (eGFR),^[17]^ were evaluated. Since inflammation and TNF-α modify iron utility as well as iron absorption and erythropoiesis,^[18,19]^ mean corpuscular hemoglobin concentrations (MCHC) were also estimated in patients treated with L/G-CAP or BP. Adverse events during the 2-year treatment period were also assessed.

### 4.3. Statistical analysis

Results are expressed as median [lower quartile-upper quartile]. Significance of differences were assessed using Mann–Whitney *U* test or Wilcoxon signed-rank test. Time-series regression analyses were applied for evaluation of temporal changes in the cumulative costs defrayed for the treatment of UC in the L/G-CAP and the BP group. Incidence of adverse events was evaluated with Fisher exact test. Statistical analyses were performed using Statistical Package for Social Sciences (SPSS) version 25 (IBM, www.ibmcom). *P* values <.05 were considered statistically significant.

## 5. Results

### 5.1. Baseline data

Table 1 shows the baseline characteristics of the patients enrolled in this study. There was no significant difference in age or body mass index (BMI) between the L/G-CAP and the BP group. CAI’s were markedly elevated in both L/G-CAP (7.0, n = 19) and BP group (10.0, n = 7, *P* = .207), in which categories the CAI’s were distributed in a similar manner (*P* = 1.0). Matts endoscopic classification (Matts score)^[20]^ showed similar levels of endoscopic activity (*P* = .248). The disease extent did not differ between the L/G-CAP and the BP group (*P* = 1.0). Furthermore, these 2 groups showed similar profiles of the responsiveness to corticosteroids (*P* = 1.0). (see Table 1).

**Table 1:** Baseline characteristics.

	L/G-CAP (n = 19)	Biological preparations (n = 7)	*P* value
Age, yr, median [IQR1–IQR3]	53.0 [46.0–65.0]	45.0 [40.0–49.0]	.083
BMI, median [IQR1–IQR3]	20.0 [18.4–23.1]	23.0 [22.8–25.2]	.072
Sex, male/female, n (%)	8/11 (42.1)	7/0 (100.0)	.010
UC duration, weeks [IQR1–IQR3]	52 [16–321]	156 [12–191]	.686
Clinical activity index (CAI), median [IQR1–IQR3]	7.0 [6.0–10.0]	10.0 [6.0–10.0]	.207
Mild, CAI = 5–6, n (%)	5 (26.4)	2 (28.6)	1.0
Moderate, CAI = 7–11, n (%)	12 (63.2)	4 (57.1)	
Severe, CAI ≥ 12, n (%)	2 (10.5)	1 (14.3)	
Matts endoscopic classification	3.0 [3.0–3.0]	2.5 [2.0–3.25]	.248
Disease extent, n (%)			
Total	10 (52.6)	4 (57.1)	1.0
Left-sided	9 (47.4)	3 (42.9)	
Response to corticosteroids, n (%)			
Resistant	5 (26.3)	2 (28.6)	1.0
Dependent	8 (42.1)	3 (42.8)	
Nonrefractory	6 (31.6)	2 (28.6)	
History of corticosteroid administration, n (%)	16 (84.2)	6 (85.7)	1.0
Concomitant medications, n (%)			
Mesalazine	18 (94.7)	6 (85.7)	.474
Corticosteroids	16 (84.2)	5 (71.4)	.588
Thiopurine	6 (31.6)	4 (57.1)	.369
Tacrolimus/cyclosporin	0 (0.0)	0 (0.0)	NA
Chronic kidney disease (eGFR < 60 mL/min/1.73 m^2^)	3 (15.8)	0 (0.0)	.540
Laboratory data, median [IQR1–IQR3]			
Leukocyte count, /mm^3^	7000 [6400–9400]	9500 [8200–12,700]	.099
Erythrocyte count, ×10^4^/mm^3^	405.0 [318.0–453.0]	408.0 [389.0–475.0]	.370
Platelet count, ×10^4^/mm^3^	29.5 [23.6–35.8]	36.7 [28.3–43.1]	.094
Hemoglobin level, g/dL	11.8 [10.1–13.3]	11.7 [9.4–13.6]	.817
CRP level, mg/dL	0.97 [0.21–2.83]	4.37 [0.50–10.11]	.060
Erythrocyte sedimentation rate, mm/h	22.0 [17.0–49.0]	59.0 [29.0–94.5]	.047

BMI = body mass index, CAI = Rachmilewitz clinical activity index, CRP = C-reactive protein, eGFR = estimated glomerular filtration rate, IQR1 = lower quartile, IQR3 = upper quartile, L/G-CAP = eukocytapheresis/granulocytapheresis, NA = not applicable, UC = ulcerative colitis.

Inflammatory markers, including leukocyte/platelet count and CRP, were moderately elevated, but no difference was noted between the L/G-CAP and the BP group for either of the parameters (leukocyte count; *P* = .099, platelet count; *P* = .094, CRP; *P* = .060, Table 1). ESR was greater in the BP group than in L/G-CAP group (*P* = .047).

### 5.2. One year treatment efficacy

**Figure 1.**
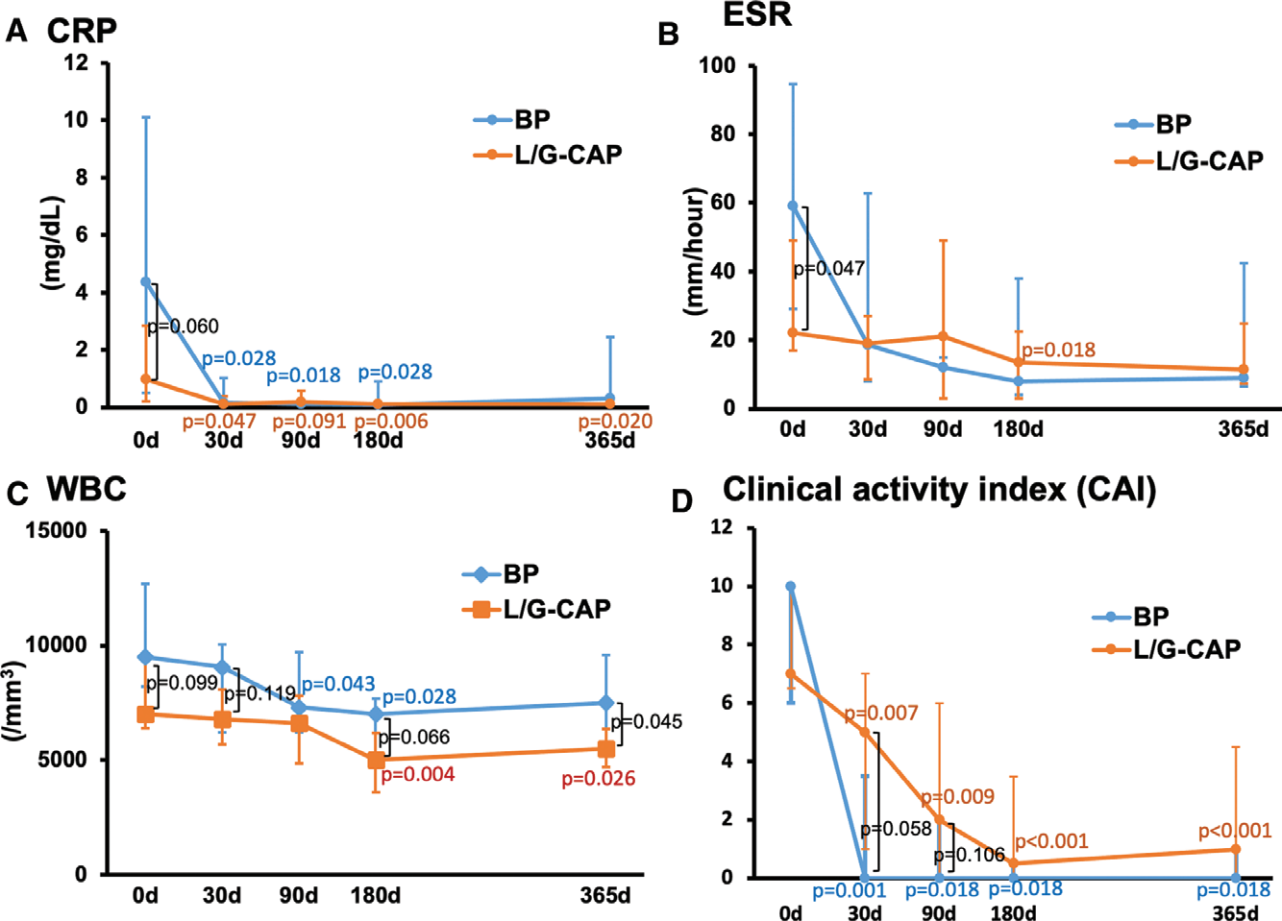
Effects of leukocytapheresis/granulocytapheresis and biological preparations on inflammatory parameters and clinical activity index in ulcerative colitis. WBC leukocyte count. Results are median [lower quartile-upper quartile]. BP = biological preparations, CRP = C-reactive protein, ESR = erythrocyte sedimentation rate, L/G-CAP = leukocytapheresis/granulocytapheresis.

In patients treated with BP, marked reductions in CRP, ESR, and CAI were observed on day 30, following which these effects were sustained throughout the 1 year study period (Fig. 1). In the L/G-CAP group, baseline levels of CRP and ESR were slightly lower, but the subsequent levels did not differ from those in the BP group. CAI was gradually suppressed and reached a level of remission but with a wide dispersion (day 180; 0.5 [0–3.5], day 365; 1.0 [0–4,5]). WBC decreased in both groups, manifesting marginally lower leukocyte counts in L/G-CAP group. (see Fig. 1).

The L/G-CAP and BP therapies ameliorated endoscopic features (Matts score, from 3.0 [3.0–3.0] to 1.5 [1.0–2.0] (*P* = .004) and from 2.5 [2.0–3.25] to 1.0 [1.0–1.25] (*P* = .041), for L/G-CAP and BP, respectively) as well as clinical symptoms (bloody stool and abdominal cramp), with nearly equal effectiveness observed in these 2 groups (data not shown).

### 5.3. Cumulative doses of steroids/mesalazine and treatment costs

**Figure 2.**
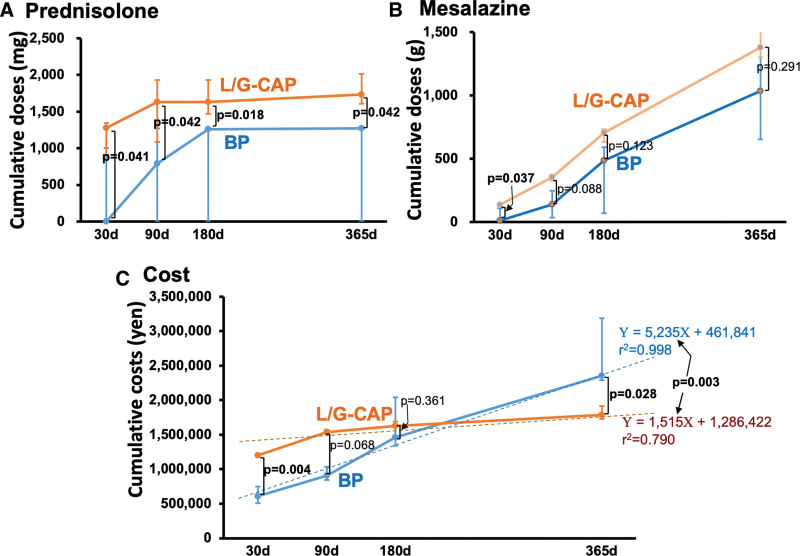
Cumulative doses of conventional medications and costs accompanying the treatment of ulcerative colitis. Prednisolone (A) and mesalazine (B) were used as conventional drugs. In the early period of the study, cumulative costs incurred for the treatment with leukocytapheresis/granulocytapheresis (L/G-CAP) were higher than those with biological preparations (BP), but were surpassed by those with BP on day 365 (C). Time-series regression analyses showed a smaller value of regression (i.e., slope) coefficient in the L/G-CAP group (i.e., α = 1515 [95%CI: -859 -+3889] vs 5235 [95%CI: 4455-6014], *P* = .003). Results are median [lower quartile-upper quartile].

Cumulative doses of prednisolone were slightly higher in the L/G-CAP than in the BP group, particularly during the early observation period (Fig. 2A); of note, the cumulative doses reached a plateau in both L/G-CAP (on day 90) and BP group (on day 180). In contrast, the cumulative dose of mesalazine was increased with time, the changes of which did not differ between these groups (Fig. 2B). (see Fig. 2).

The cumulative cost defrayed for the treatment with L/G-CAP plus baseline drugs (e.g., mesalazine and corticosteroids) was higher than that with BP when evaluated at 30 days from the initiation of the therapy (L/G-CAP; 1197,925 [1185,108–1199,476] yen, BP; 610,466 [503,696–748,008] yen, *P* = .004, Fig. 2C). On 180 days, however, the cost in the L/G-CAP group was nearly equal to that in the BP group (*P* = .361). Over 365-day period, the cost entailed in the BP group amounted to 2352,622 [2291,493–3187,826] yen. The expenditure for the treatment with L/G-CAP, by contrast, was curtailed to 1787,450 [1727,466–1915,254] yen, a value 24% less than that with BP (*P* = .028). Time-series regression analysis for cumulative costs showed that the regression coefficient for the L/G-CAP group was markedly lower than that for the BP group (*P* = .003).

### 5.4. Evaluation of 2-year sustained efficacy of L/G-CAP

**Figure 3.**
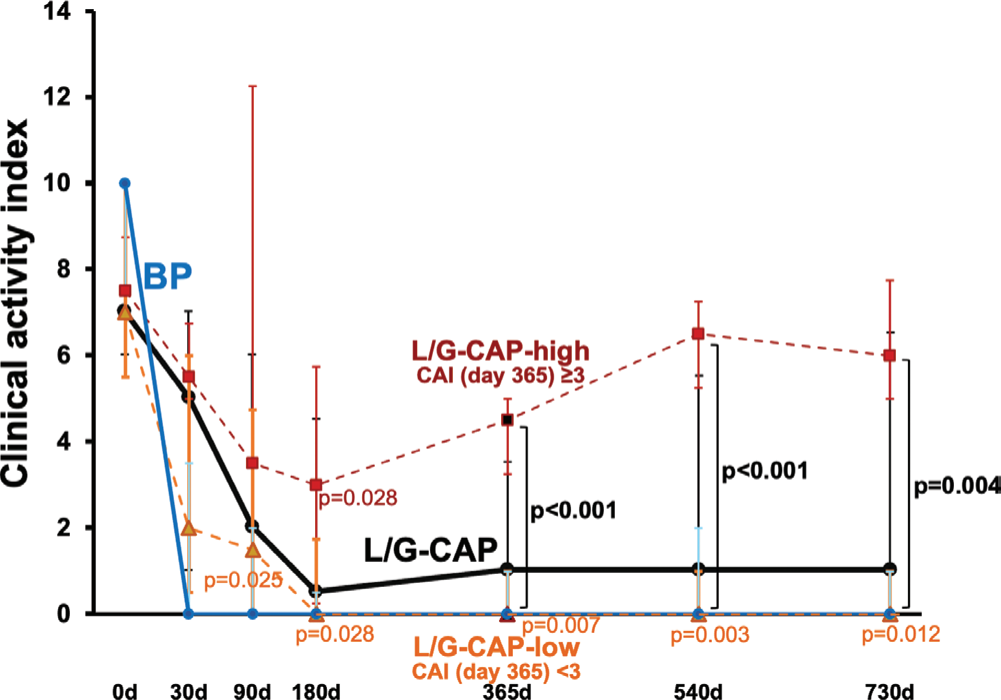
Effects of leukocytapheresis/granulocytapheresis and biological preparations on clinical activity index over 2 years. Patients treated with leukocytapheresis/granulocytapheresis (L/G-CAP) or biological preparations (BP) manifested striking reductions in clinical activity index (CAI) although the responses in the L/G-CAP group were widely dispersed. Hence, patients treated with L/G-CAP were further divided into 2 subgroups, depending on the CAI value on day 365 (CAI ≥ 3; L/G-CAP-high, CAI < 3; L/G-CAP-low). Results are median [lower quartile-upper quartile].

We next assessed whether a series (i.e., 10 sessions) of the L/G-CAP therapy offered sustained beneficial effects on suppressing the UC activity over 2 years. Thus, CAI remained suppressed but with wide dispersions on day 540 (1.0 [0–5.5]) and day 730 (1.0 [0–6.5], n = 19, Fig. 3). In the BP group, the treatment was continued with the same protocol as before, and the responses were found stabilized in remission throughout the study period. (see Fig. 3).

To further characterize the temporal changes in CAI in the L/G-CAP group, this group was divided into 2 subgroups according to the CAI values on day 365 [L/G-CAP-high; CAI ≥ 3 (n = 8) and L/G-CAP-low; CAI < 3 (n = 11)] (Fig. 3). Matts endoscopic scores also showed different profiles between the L/G-CAP-high subgroup (from 3.0 [3.0–3.25] to 2.0 [1.75–2.25] on day 365, *P* = .102) and L/G-CAP-low subgroup (from 3.0 [3.0–3.0] to 1.0 [1.0–1.75] on day 365, *P* = .024).

At baseline (i.e., day 0), all anthropometric parameters (age, BMI) as well as the disease activity were nearly the same between these 2 subgroups (Table 2). Likewise, there was observed no difference in blood hemoglobin levels between these subgroups. (see Table 2).

**Table 2:** Baseline results in the L/G-CAP group with high or low CAI on day 365.

Baseline data	CAI on day 365		*P* value
	≥3 (n = 8) [L/G-CAP-high]	<3 (n = 11) [L/G-CAP-low]	
Age (y/mo)	53.0 [48.8–62.0]	61.0 [50.0–64.0]	.401
BMI (kg/m^2^)	21.0 [19.3–22.8]	19.6 [18.2–23.3]	.331
Sex, male/female, n (%)	4/5 (42.1)	5/6 (100.0)	1.0
UC duration (weeks)	38 [21–77]	104 [44–530]	.264
Clinical activity index (CAI)	7.5 [7.0–8.75]	7.0 [5.5–10.0]	.460
Matts endoscopic classification	3.0 [3.0–3.25]	3.0 [3.0–3.0]	.350
Laboratory data			
Hemoglobin, g/dL	12.2 [10.5–13.1]	11.6 [9.8–12.8]	.257
MCHC, g/dL	29.2 [28.0–29.4]	30.7 [29.2–33.2]	.035
CRP, mg/dL	0.99 [0.39–1.73]	1.11 [0.17–3.45]	.256
Erythrocyte sedimentation rate, mm/h	22 [21–42]	22 [17–59]	.421

BMI = body mass index, CAI = Rachmilewitz clinical activity index, CRP = C-reactive protein, L/G-CAP = eukocytapheresis/granulocytapheresis, MCHC = mean corpuscular hemoglobin concentration, UC = ulcerative colitis.

In the L/G-CAP-high subgroup, the apheresis-induced suppression of CAI tended to be abated compared with the L/G-CAP-low subgroup, and significantly attenuated effects were observed at 365 days (*P* < .001), 540 days (*P* < .001) and 730 days (*P* = .004, Fig 3). In contrast, markedly favorable responses to the apheresis therapy were observed in the L/G-CAP-low subgroup, which persisted throughout the study period.

**Figure 4.**
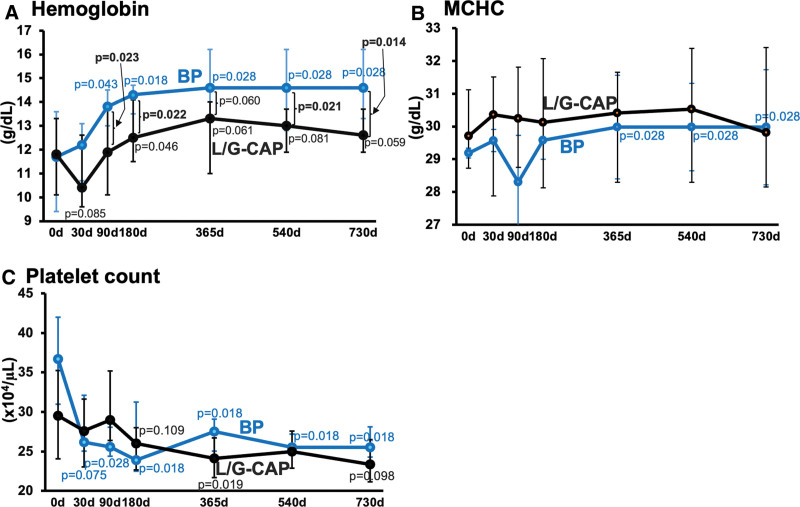
Two-year changes in hemoglobin, mean corpuscular hemoglobin concentration, and platelet counts in patients with ulcerative colitis treated with leukocytapheresis/ granulocytapheresis or biological preparations. Results are median [lower quartile-upper quartile]. BP = biological preparations, L/G-CAP = leukocytapheresis/granulocytapheresis, MCHC = mean corpuscular hemoglobin concentration.

The treatment with BP markedly increased blood hemoglobin levels, producing significant improvements on day 90 (*P* = .043), and the response was sustained throughout the study period (Fig. 4A). In the L/G-CAP group, a modest decrease (on day 30) followed by a slight elevation in blood hemoglobin was observed (on day 180), though the subsequent responses did not attain statistical significance. In the L/G-CAP-low subgroup, significant increases in hemoglobin were seen from 11.6 [9.75–12.8] g/dL on day 0 to 13.3 [11.4–13.8] g/dL on day 365 (*P* = .029), to 12.4 [12.2–13.7] g/dL on day 540 (*P* = .014) and to 12.6 [12.2–13.7] g/dL on day 730 (*P* = .032), but not in the L/G-CAP-high subgroup. The BP therapy caused a significant elevation in MCHC on day 365 (from 29.2 [28.1–29.3] to 30.0 [29.3–31.7] g/dL, *P* = .028) and thereafter (Fig. 4B). In contrast, L/G-CAP had no effect on MCHC. (see Fig. 4).

The BP therapy markedly decreased platelet count on day 30, which response was sustained throughout the observation period (Fig. 4C). In the L/G-CAP group, a modest reduction was observed on day 365 (*P* = .019).

In response to BP, eGFR decreased from 83.2 [78.8–89.6] to73.5 [70.1–85.0] mL/minutes/1.73 m^2^ on day 90 (*P* = .023) and was almost unaltered thereafter (Figure S1A, Supplemental Digital Content, http://links.lww.com/MD/I712). In the L/G-CAP group, eGFR was reduced from 82.5 [64.0–93.8] to 71.9 [62.0–79.5] mL/minutes/1.73 m^2^ on day 365 (*P* = .004) but no further changes were seen over the next year. BMI tended to increase in the BP group (*P* = .100) but was unaltered in the L/G-CAP group during the 1 year period (Figure S1B, Supplemental Digital Content, http://links.lww.com/MD/I712). Similarly, serum creatinine concentrations were elevated in the BP and marginally increased in the L/G-CAP group (Figure S1C, Supplemental Digital Content, http://links.lww.com/MD/I712). When evaluated among the whole populations, including the BP and the L/G-CAP group, a trend toward a positive correlation was observed between the changes in BMI and those in serum creatinine concentrations (r^2^ = 0.333, *P* = .081). (see Figure S1, Supplemental Digital Content, http://links.lww.com/MD/I712).

### 5.5. Adverse events

In the L/G-CAP group, an elevation in body temperature (1 case) and skin rash (2 cases) were observed, both of which disappeared spontaneously (Table 3). Other apheresis-associated adverse events, including abdominal pain, headache, respiratory distress and a decrease in platelet count, eventually subsided with no additional therapy. (see Table 3).

**Table 3:** Adverse events during the treatment with L/G-CAP or biological preparations.

	L/G-CAP (n = 19)	Biological preparations (n = 7)	*P* value
General disorders and administration site conditions			
Fever	1	1	.474
Chill	0	0	NA
Pain in vascular access site	0	0	NA
Gastrointestinal disorders			
Nausea	0	0	NA
Abdominal pain	1	0	1.0
Nervous system disorders (Headache)	1	0	1.0
Circulatory disorders (hypotension)	0	0	NA
Dermatological disorders (skin rash)	2	0	1.0
Respiratory disorders			
Nasal congestion	0	0	NA
Respiratory distress	1	0	1.0
Kidney disease			
Acute kidney injury	1	1	.474
New onset of chronic kidney disease	0	1	.269
Immune system disorders			
Anaphylactic shock	0	0	NA
Infectious disorders	0	1	.269
Sepsis	0	0	NA
Laboratory data abnormalities			
Platelet count decrease	1	0	1.0
Liver enzymes increase	0	0	NA

L/G-CAP = leukocytapheresis/granulocytapheresis, NA = not applicable.

In the BP group, no serious infection or infusion-site reaction associated with anti-TNF-α antibody agents occurred although 1 patient manifested fever and infectious disease (i.e., transient bacteremia due to bacterial translocation, Table 3).

Acute kidney injury occurred in the early period of the study in both L/G-CAP and BP groups (1 case for each group, Table 3), which was recovered by appropriate treatment, including volume replacement therapy. Although 1 patient in the BP group manifested a new onset of chronic kidney disease, the renal function did not deteriorate further but was stabilized over 1 year (eGFR, 51 mL/minutes/1.73 m^2^).

## 6. Discussion

### 6.1. One year evaluation of efficacy of L/G-CAP

The present retrospective study shows that L/G-CAP suppresses the disease activity in moderately to severely active UC as effectively as BP during the 1 year period after the implementation of L/G-CAP. Thus, these 2 modalities improved the biochemical parameters pertaining to inflammation in UC patients (Fig. 1), most of whom had precedently been on conventional drugs (mesalazine, corticosteroids or immunomodulators); nearly complete suppression of CRP was seen as early as 30 days after initiation of the treatment. Furthermore, both treatment strategies markedly ameliorated the clinical activity of UC and the symptoms such as abdominal cramp and general condition. Evidence has accrued that L/G-CAP removes excess and activated leukocytes from the peripheral blood and inhibits the inflammation of the colonic mucosa.^[8–10]^ Similarly, BP’s, including monoclonal antibody agents against TNF-α and α4β7-integrin and JAK inhibitors, potently induce clinical remission by abating inflammation and inhibiting inflammatory cytokine-mediated signal transduction.^[1,5,21,22]^ It appears therefore that whichever therapeutic tool is adopted, elimination of the inflammatory response at the colonic mucosa constitutes a bottom-line strategy for alleviating the activity of UC.

The role of new strategies in the treatment of UC is rapidly growing. A number of systematic reviews and meta-analyses show that anti-TNF-α monoclonal antibody agents are more effective than conventional drugs,^[6,13,23,24]^ and are ranked highest as first-line agents for moderately to severely active UC. Likewise, there reported are a substantial number of multicenter studies^[14,25]^ and open label trials^[26,27]^ demonstrating beneficial effects of L/G-CAP therapies. Nevertheless, there have so far been no studies comparing the efficacy of L/G-CAP and BP. Although the present study is not intended for making a direct head-to-head comparison of the effects of these 2 treatment strategies, both therapies appear to be equally effective when evaluated in moderately to severely active UC patients for 1 year (Fig. 1).

### 6.2. Cost-effectiveness of L/G-CAP versus BP

Despite the allegedly similar effectiveness of L/G-CAP and BP in the treatment of UC with moderate to severe activity, the issue of the costs pertaining to these therapies would constitute a focus of debate.^[12,13,28]^ As acknowledged generally, BP is much more expensive than conventional drugs.^[13,28]^ A current protocol for UC therapies indicates that the maintenance therapy with BP should be continued even though the disease activity stays in remission, whereby the cumulative cost would constantly rise with time at a high rate (Fig. 2C). Likewise, L/G-CAP requires extracorporeal devices and cell adhesion columns, and hence incurs more expenses than conventional drug therapies.^[13]^ In the current study, however, we conducted no additional session after an initial series of L/G-CAP. Thus, although the cumulative costs entailed with L/G-CAP were higher or tended to be more expensive than those with BP during the early observation period (30–90 days from the initiation of therapy), the cost over 1 year was rather less in the L/G-CAP group. Although the cumulative dose of prednisolone was slightly higher in the L/G-CAP group, it did not affect the incidence of adverse events nor the cost entailed with these therapies (Fig. 2, Table 3). Provided that the treatment policy for each group remains unaltered, it may fairly be inferred that the cost-effectiveness is in favor of L/G-CAP rather than BP when assessed over 1 year or possibly longer periods.

### 6.3. Sustained efficacy of L/G-CAP

Although the present study shows a beneficial effect of L/G-CAP on suppressing the disease activity of UC, there exists a wide dispersion of CAI’s, particularly at 365 days and thereafter (Fig. 3). We therefore divided this group into 2 subgroups (L/G-CAP-high and -low), depending on the CAI value (≥3 or < 3,^[14]^) on day 365. Thus, a striking difference in the temporal changes of CAI, along with Matts endoscopic scores, was noted between these subgroups. Although we remain uncertain as to whether this criterion (i.e., CAI ≥ 3 or < 3 on day 365) is more appropriate to group dichotomization, several other possibilities appear less clear-cut (Figure S2, Supplemental Digital Content, http://links.lww.com/MD/I713). Thus, there are a substantial number of cases who respond well to L/G-CAP and have long-lasting (i.e., 2 years) remission without additional implementation of this procedure. Indeed, Kobayashi et al^[14]^ showed that the majority of patients (>60%) treated with L/G-CAP achieved clinical remission and remained relapse-free for 1 year. Furthermore, Takayama et al^[15]^ demonstrated that among the UC patients followed for more than 3 years, approximately half of the UC patients treated with L/G-CAP obtained clinical remission after 1 course of L/G-CAP treatment. They also show that patients who respond to the first course of L/G-CAP will respond to the second series of this procedure. In concert, clear-cut criteria would yield cost-effective therapeutic strategy whereby the apheresis offers long-lasting benefit. (see Figure S2, Supplemental Digital Content, http://links.lww.com/MD/I713).

Anemia is one of the most common complications of UC and is attributed to chronic inflammatory process as well as iron deficiency.^[18,19]^ The present study shows that blood hemoglobin levels rise shortly after the treatment with BP, compared with the response to L/G-CAP (Fig. 4A). This favorable effect may be accounted partly for by the rapid suppression of inflammation by BP (Figs. 1 and 3). Thus, both infliximab and adalimumab act directly against TNF-α to suppress inflammatory process and are reported to improve iron metabolism through modulation of a cytokine network involving hepcidin and IL-6 [18]. Of interest, MCHC, a possible marker for iron availability during erythropoiesis,^[29,30]^ is elevated by BP, but not by L/G-CAP (Fig. 4B). In contrast, L/G-CAP, which methodologically requires extracorporeal circulation, might more or less decreases blood hemoglobin levels^[31,32]^ though it also acts to improve anemia through stabilization of the disease activity. Similarly, BP corrected thrombocytosis more rapidly than L/G-CAP (Fig. 4C). Platelet count is commonly elevated in active inflammatory bowel disease,^[33,34]^ the mechanisms of which are thought to be mediated by several proinflammatory cytokines, including IL-6 and TNF-α.^[18,35]^ In concert, alleviation of inflammatory processes contributes to the improvement in blood abnormalities, which varies in time course and efficacy depending on the treatment modalities used. Whether the rapid correction of the blood abnormalities offers substantial benefits on a long-term basis awaits further evaluation.

The present study shows that BP elicits an initial fall in eGFR but neither BP nor L/G-CAP treatment causes a further reduction in eGFR (Figure S1A, Supplemental Digital Content, http://links.lww.com/MD/I712). Although mesalazine is reported to cause interstitial nephritis, the stable temporal course of eGFR observed on day 180 and thereafter rather eliminates this possibility.^[36]^ In this regard, Mahmud et al^[37]^ showed that 9-month treatment with mesalazine had no significant effect on GFR estimated by technetium-labeled DPTA. Alternatively, the disease activity per se may cause tubulointerstitial damage as an extraintestinal complication.^[38,39]^ Finally, the mitigated disease activity induced by BP would improve nutritional status (Figure S1B, Supplemental Digital Content, http://links.lww.com/MD/I712) and might potentially affect creatinine metabolism through changes in muscle bulk,^[40,41]^ which could result in elevated serum creatinine levels (Figure S1C, Supplemental Digital Content, http://links.lww.com/MD/I712) and the nominal reduction in eGFR. It needs to be clarified whether renal manifestation of UC and renal effects of the therapeutic tools have long-term impact on renal function.

## 7. Limitations

There are several limitations posited in the present study. Our retrospective study was conducted in 2 individual facilities for relatively a short duration, and the study population was small. The results from this study therefore need to be scrutinized before extrapolation of our findings to a general population. Furthermore, current treatment protocols for UC with anti-TNF-α monoclonal antibody agents stick in principle to the continued use of these products even though the remission is maintained. In contrast, the L/G-CAP therapy is conducted at the start of the study and no additional session of L/G-CAP is performed unless required, which strategy might tend to elevate the disease activity in a certain population. Because these treatment protocols could be modified to accommodate the therapeutic strategies to real-world medical therapy, further investigations would improve the cost-effectiveness in the treatment of moderately to severely active UC.

## 8. Conclusions

Therapeutic strategies for moderately to severely active UC are unremittingly progressing, with novel biological products and apheresis devices being developed and available in clinical practice. Alternatively, growing concern arises regarding the costs of new biological products and apheresis modality, which therefore will necessitate the cost-effectiveness analysis in the treatment of UC. More simplified protocols for the treatment with BP and characterization of a specific population with sustained suppression of UC activity induced by the cytapheresis could elucidate the well-balanced cost-effectiveness in the treatment of UC.

## Author contributions

**Conceptualization:** Masahiro Sakai, Shigeki Fujitani, Toshihiko Suzuki.

**Data curation:** Masahiro Sakai, Tomoyuki Ito, Haruka Otani, Yuya Mori, Keita Endo, Hiroto Matsuda, Eiji Kubota, Yasuaki Motomura.

**Formal analysis:** Masahiro Sakai, Koichi Hayashi, Shinsuke Ito.

**Investigation:** Masahiro Sakai, Kaede Yoshino, Koichi Kitamura.

**Methodology:** Masahiro Sakai, Yasuhiro Suzuki.

**Project administration:** Koichi Hayashi, Toshihiko Suzuki.

**Supervision:** Shigeki Fujitani, Toshihiko Suzuki.

**Writing – original draft:** Masahiro Sakai.

**Writing – review & editing:** Koichi Hayashi, Shigeki Fujitani, Toshihiko Suzuki.

## Supplementary Material




