# Supplementary material for: Sustained effect of leukocytapheresis/granulocytapheresis versus anti-human TNF-α monoclonal antibody on ulcerative colitis: A 2-year retrospective study
Source: Medicine (Baltimore). 2023 Apr 21;102(16):e33368. doi: 10.1097/MD.0000000000033368 (PMC10118317; doi:10.1097/MD.0000000000033368)

Supplemental figure 1. Changes in eGFR, body mass index and serum creatinine concentrations.

Following the treatment with BP or L/G-CAP, eGFR was initially decreased and then stabilized after 90 days and 180 days, respectively (A). Body mass index (BMI) tended to increase in the BP group at 365 days (B). Serum creatinine concentrations were significantly elevated in the BP, but not L/G-CAP, group (C). BP; biological preparations, L/G-CAP; leukocytapheresis/granulocytapheresis.

Supplemental figure 1.

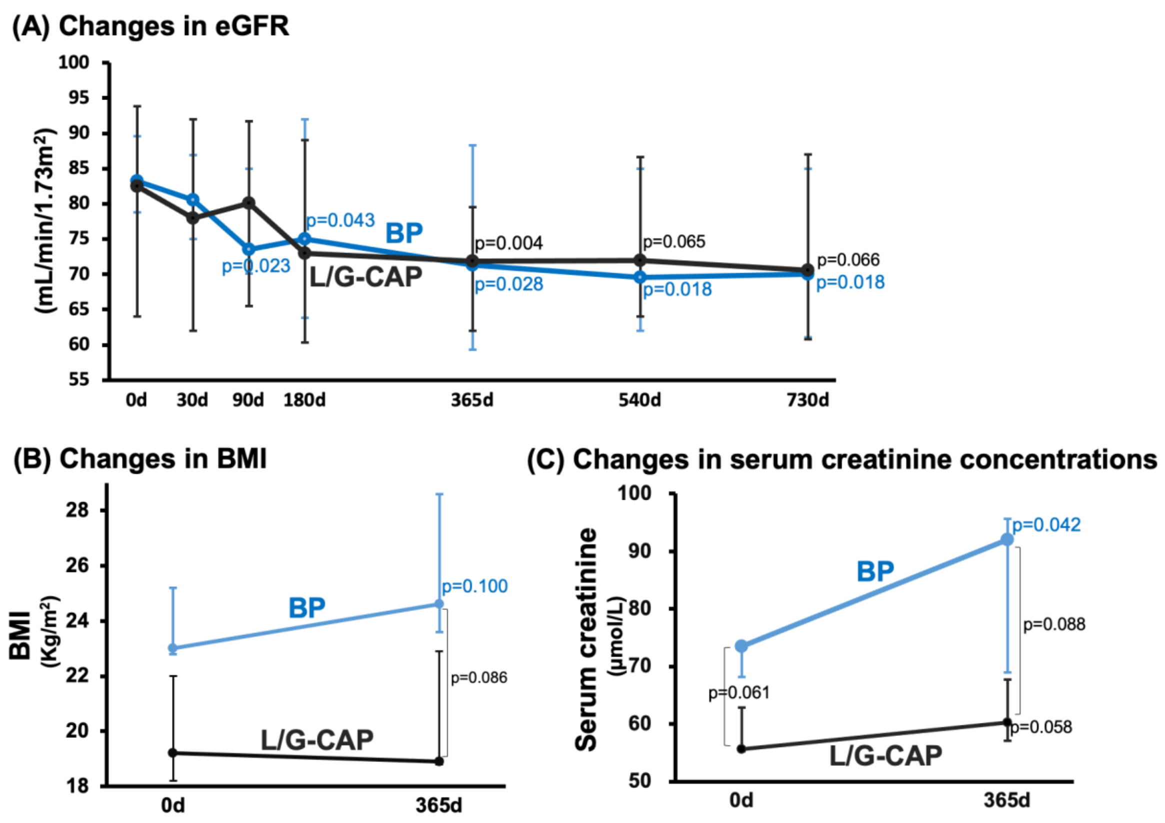

Supplement: Supplementary file 1 [file medi-102-e33368-s001.pdf]
