# Supplementary material for: Sustained effect of leukocytapheresis/granulocytapheresis versus anti-human TNF-α monoclonal antibody on ulcerative colitis: A 2-year retrospective study
Source: Medicine (Baltimore). 2023 Apr 21;102(16):e33368. doi: 10.1097/MD.0000000000033368 (PMC10118317; doi:10.1097/MD.0000000000033368)

Supplemental figure 2. Dichotomization of leukocytapheresis/granulocytapheresis group.

The patient group treated with leukocytapheresis/granulocytapheresis was divided into two subgroups based on the clinical activity index (CAI, ≥3 or <3) observed on specific days (i.e., on 30, 90, 180, 365 or 540 days) and the results were analyzed in each categorization.

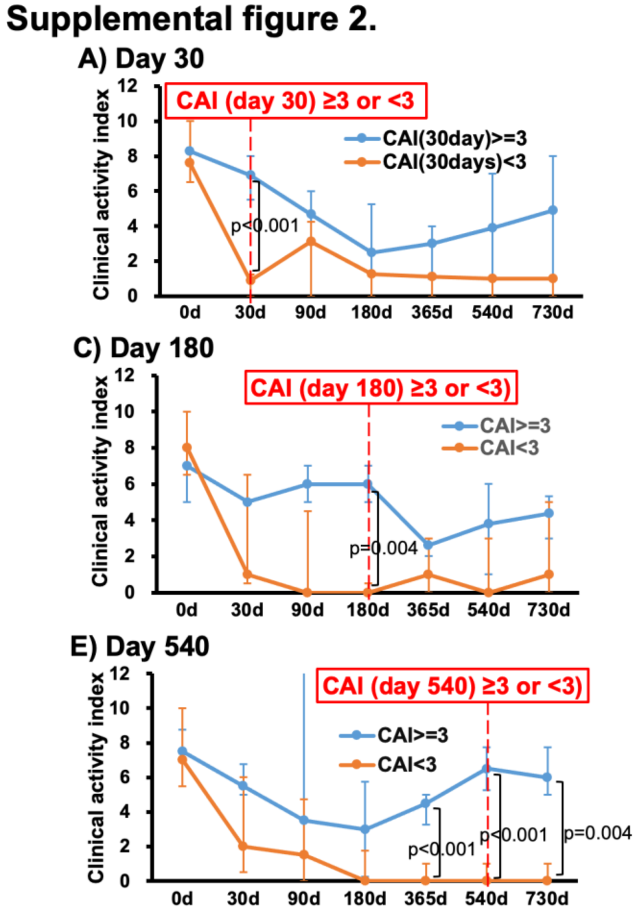

Supplement: Supplementary file 2 [file medi-102-e33368-s002.pdf]
